# Digit Ratio (2D:4D) Is Not Associated with Alzheimer’s Disease in the Elderly

**DOI:** 10.3390/brainsci13091229

**Published:** 2023-08-22

**Authors:** Eva-Maria Siegmann, Pauline Olm, Bernd Lenz, Christiane Mühle, Timo Jan Oberstein, Juan Manuel Maler, Johannes Kornhuber

**Affiliations:** 1 Department of Psychiatry and Psychotherapy, Friedrich-Alexander-Universität Erlangen-Nürnberg (FAU), Schwabachanlage 6, 91054 Erlangen, Germany; 2 Department of Addictive Behavior and Addiction Medicine, Central Institute of Mental Health (CIMH), Medical Faculty Mannheim, Heidelberg University, 68159 Mannheim, Germany

**Keywords:** Alzheimer’s disease, digit ratio, dementia, fetal development, finger length ratio

## Abstract

The development of Alzheimer’s disease (AD) is influenced by sex hormones—estrogens and androgens in particular. However, the impact of prenatal sex hormone exposure is less clear; very few investigations have examined the relationship between the second-to-fourth digit length ratio (2D:4D), a putative proxy for the ratio of prenatal estrogens to androgens, and AD, with inconsistent results among the few that have. Therefore, we aimed to investigate this relationship using methodologically robust metrics. In a 2 (sex) × 4 (group) MANOVA incorporating 108 participants (30 AD patients, 19 patients with tauopathy but no amyloidopathy, 31 clinical and 28 healthy age- and education-matched controls), the effects of sex and group on the dependent variables right and left 2D:4D were examined. We also explored the association between 2D:4D and the severity of AD symptoms assessed via neuropsychological examination. We did not find any significant differences in the right- and left-hand 2D:4D between patients with AD and the other groups; no significant associations between 2D:4D and neuropsychological task performances were found in the dementia groups. The 2D:4D of healthy women was significantly lower than that of depressed women without AD, i.e., clinical controls, but not significantly different from depressed female patients with AD. This investigation does not support the role of 2D:4D in the development or severity of AD in general, but suggests a potential role of 2D:4D for depression in women. Future studies are warranted to clarify whether 2D:4D can distinguish between early- and late-onset depression in women.

## 1. Introduction

Alzheimer’s disease (AD) is a neurodegenerative disorder characterized by amyloidopathy (lower levels of amyloid beta [Aβ] 42 or lower ratio of Aβ42 to Aβ40) and tauopathy (elevated levels of phosphorylated tau [pTau]) in the cerebrospinal fluid (CSF)) [1,2,3]. The appearance of AD differs between men and women regarding prevalence rates [4], symptom progression and vulnerability to the disease [5]. Sex hormone levels in adulthood associate with AD sex-specifically: in males, lower levels of testosterone are related to a greater risk of developing AD [6] and higher levels of testosterone predict better cognitive performance later in life [7]. Moreover, male patients undergoing androgen deprivation therapy exhibit a higher incidence of AD [8]. This is supported by an animal study indicating that the more feminized (i.e., demasculinized by treatment with androgen receptor antagonist flutamide) a male mouse is, the more likely it is to develop AD [9]. In females, a correlative relationship between low estrogen and AD is proposed by multiple studies (for a summary of those studies, see the detailed reviews by Pike (2017) [5] and Hogervorst and Bandelow (2007) [10]). This is further confirmed in animal studies showing that ovariectomy in rodents leads to elevated levels of soluble Aβ [11]. Altogether, sex-matching levels of estrogens and androgens seem to be neuroprotective against the accumulation of Aβ in both men and women [12].

In addition to the direct influences of sex hormone levels on the brain, prenatal sex hormone exposure modulates human brain organization and behavior as well [13,14,15,16,17,18]. A direct measurement of prenatal sex hormone levels in humans in experimental studies, however, is not possible due to ethical and practical reasons. Therefore, the second-to-fourth digit length ratio (2D:4D) is often used as a proxy for prenatal androgen load [19,20,21], i.e., 2D:4D is said to reflect individual differences in prenatal exposure to sex hormones [22]. It is determined by a high ratio of prenatal testosterone to estradiol [23,24,25], with a lower 2D:4D reflecting a higher prenatal androgen exposure. Thus, 2D:4D is lower in males than in females [20,26,27]. This assumption relies among others on evidence linking 2D:4D with medical conditions that affect endocrinological pathways (e.g., congenital adrenal hyperplasia (CAH) [28], Klinefelter syndrome [29,30] and complete androgen insensitivity syndrome (CAIS) [31]) or with amniotic testosterone, i.e., actual hormone levels during the prenatal period [22,24,32]. A lower 2D:4D associates, for example, with addictive disorders [33,34,35,36,37,38], autism spectrum disorders [39,40] and sexual orientation or identity [41,42].

Studies on the direct relationship between 2D:4D and cognitive performance or AD are rare and inconclusive: Barel and Tzischinsky (2017) [43] suggested an interaction between 2D:4D and sex in memory task performance, insofar that for males this association is negative with a lower 2D:4D (indicative of higher prenatal androgenization), relating to higher cognitive functioning, while in females no significant differences were observed. In contrast, most other studies in the field describe significant findings for females. Goncalves et al. (2017) [44] found a significant positive correlation with the Mini-Mental State Examination (MMSE) score [45] in women, suggesting a preventive effect of low androgen exposure in utero for females. In a magnetic resonance imaging (MRI) study of healthy women, the volume of the left posterior hippocampus, a brain region often atrophic in AD [46], was lower in females with masculinized 2D:4D [47]. Studies on differences in 2D:4D among AD patients compared with controls have revealed both higher 2D:4D [48] and lower 2D:4D [49,50] in female AD patients, as well as feminized 2D:4D in male AD patients [49]. However, the generalizability of these studies is limited, as they report pilot data [49] or abstract data [50] and use self-report criteria to allocate cases versus controls [48]. An additional difficulty arises from the definition of control subjects in this research field. People without dementia can nevertheless suffer from other psychiatric illnesses, as those are widespread in the general population [51,52]. Some studies report that 2D:4D is altered in psychiatric patients, for example, in depression [53,54,55]. Depressive symptoms, in turn, are prevalent in people with dementia [56,57] and may appear similar to AD regarding concentration difficulties and memory loss (so-called pseudo-dementia) [58]. Therefore, it is important to consider this potential confounder when designing a study on 2D:4D and AD.

The objective of the present study was to examine the role of 2D:4D in patients with confirmed AD diagnosis. In clinical practice, a combination of CSF analysis, cerebral imaging methods and neuropsychological examinations is used to diagnose AD [59]. This provides a clear distinction between AD and other neurodegenerative disorders such as Lewy body, frontotemporal and vascular dementias [1,60,61]. However, it remains unclear how to categorize subjects with tauopathy in the absence of amyloidopathy; originally, high pTau 181 levels were classified as AD-specific [62], but more recent research suggests that subjects with tauopathy only might not be on the AD spectrum [63,64]. Therefore, we investigated differences in the 2D:4D of patients with confirmed AD compared with patients with tauopathy diagnoses and with control subjects. The controls were divided into healthy subjects without any psychiatric or neurological disease and into clinical controls reporting psychiatric symptoms while a neurodegenerative diagnosis had been ruled out. Based on the literature, we hypothesized that the 2D:4D of female patients with AD is lower than that of female controls and that the 2D:4D of male patients with AD is higher than that of male controls. We followed an exploratory approach when comparing the tauopathy patients to all other groups. We further investigated performance in the neuropsychological examination and in different memory tasks associated with the 2D:4D of AD and tauopathy patients, for which we also followed an exploratory approach on this matter.

## 2. Materials and Methods

### 2.1. Sample Description

The participants were recruited from July 2020 until October 2021 at the Department of Psychiatry and Psychotherapy at the University hospital Erlangen, Friedrich-Alexander University, Erlangen-Nürnberg, Germany. The patients (i.e., AD, tauopathy or clinical control group) were enrolled in the study during their routine diagnostic assessments (either first contact or follow-up contact with the clinic) due to a (subjective) memory impairment. The detailed recruitment process of the study from which we chose to examine a subset of participants (inclusion criteria, see below) is described by Oberstein et al. (2022) [64] and Haas et al. (2023) [65]. To recruit healthy controls, we asked the patients’ spouses or former healthy control subjects from other scientific studies in the department. After a short telephonic screening assessing potential exclusion criteria, eligible healthy controls were examined at the Department of Psychiatry and Psychotherapy at the University hospital Erlangen as well. All participants underwent a hand scan (for determination of 2D:4D), a neuropsychological examination (or records were used if from within the last 6 months), a psychological assessment and—except for the healthy controls—a lumbar puncture providing complete data on Aβ42, the ratio of Aβ42 to Aβ40, pTau 181 and total Tau (tTau) in the CSF. CSF biomarkers were analyzed with ELISA provided by Fujirebio (Tokyo, Japan) (pTau 181 and tTau) and by IBL International GmbH (Hamburg, Germany) (Aβ42 and Aβ40), which are validated tools in the diagnostics of AD [66]. The patients’ group allocation was conducted according to the following validated criteria [1,67]: In the CSF of AD patients, Aβ42, the ratio of Aβ42 to Aβ40, pTau 181 and (optionally) tTau were out of the norm; In the CSF of tauopathy patients, pTau 181 and (optionally) tTau were out of the norm, while Aβ42 and the ratio of Aβ42 to Aβ40 were normal. The CSF biomarkers of clinical controls were normal, while they were suffering from a psychiatric condition diagnosed by a psychiatrist and their neuropsychological examination indicated a pseudo-dementia typical of depressive patients. Exclusion criteria were age younger than 50 years, other neurological illnesses apart from AD (e.g., confluent microangiopathy or stroke), arthritis or previous fractures of the second or fourth digits on both hands, hormone influencing factors (e.g., CAH, androgen resistance, post-menopausal hormone replacement therapy) and difficulties making a clear diagnosis. Additionally, healthy controls were excluded due to psychiatric or psychological treatment for a mental disorder in the last 10 years and conspicuous scores in the (neuro-)psychological assessments.

In an a priori power analysis, the ideal sample size was computed using the program GPower, version 3.1.9.4 [68]. Based on the pilot analysis by Vladeanu et al. (2014) [49]—up to our knowledge the only published study investigating 2D:4D and AD during the phase of study design in 2019—large effect sizes would be expected. However, this seems to be an unrealistic expectation in the context of 2D:4D research, where meta-analyses mostly suggest small to medium effects (e.g., [33,39,42]). The meta-analytically determined sex difference in R2D:4D between males and females [27] (Cohen’s d = 0.457, convertible to η^2^ = 0.05) might serve as an orientation of an expectable (i.e., a more realistic) effect size. To reduce α-error accumulation and to increase the power of our analysis, we decided to compute a 2 (sex) × 4 (group) multivariate analysis of variance (MANOVA) with two dependent variables (R2D:4D, L2D:4D). Therefore, the following parameters were combined: an expected effect size of f^2^ = 0.06 (calculated from η^2^ = 0.05), an α level of 0.05, a power of 1 − β = 0.8, 4 groups, 2 predictors and 2 response variables, resulting in an ideal sample size of 102 participants in total.

### 2.2. Neuropsychological Examination and Psychological Assessment

All participants underwent a recent neuropsychological examination (≤6 months) conducted by an experienced geriatric psychologist (P.O.) using the German version of the CERAD Plus neuropsychological battery (CERAD-NB+) [69]. This tool assesses typical difficulties in patients with AD (dyspraxia, disorientation, memory difficulties and restraints in receptive and expressive language) and is able to distinguish between different stages of dementia as well as pseudo-dementia [58]. According to Barth et al. (2005) [58], the subtests Word List Memory, Word List Recall, Constructional Praxis Recall and Phonematic Verbal Fluency Test are the most indicative of differences between people with dementia, depressive subjects and healthy subjects; therefore, we focused on these four tests and the MMSE in our analysis. The psychological assessment tools are detailed in the Supplementary Methods.

### 2.3. Second-to-Fourth Digit Length Ratio

The participants’ right and left hands were scanned on a common document scanner following the procedure established by Kornhuber et al. (2011) [70]. The lengths of the second (2D) and fourth (4D) digits were measured from the middle of the basal crease to the tip of the finger using ImageJ, version 1.53f51 [71], three times each by three independent raters. Two of the raters, both research assistants (M.S., J.G.), were blind to the study design, hypotheses, sex and the subjects’ group allocation; one rater (E.S.) was blind to sex and group allocation. The raters’ scores were averaged and 2D:4D values were calculated by dividing the length of 2D by the length of 4D for the right (R2D:4D) and the left (L2D:4D) hands. We found high interrater reliabilities (two-way random effects intraclass correlation coefficient (ICC), absolute agreement, mean of three raters with confidence interval [CI]) for the finger measurements and digit ratios: (1) Right 2D: ICC = 0.99 (95% CI [0.98; 0.99]); (2) right 4D: ICC = 0.98 (95% CI [0.97; 0.99]); (3) left 2D: ICC = 0.99 (95% CI [0.98; 0.99]); (4) left 4D: ICC = 0.98 (95% CI [0.96; 0.99]); (5) R2D:4D: ICC = 0.85 (95% CI [0.80; 0.89]); (6) L2D:4D: ICC = 0.84 (95% CI [0.79; 0.88]).

### 2.4. Ethical Standards

The study was approved by the Ethics Committee of the Medical Faculty of the Friedrich-Alexander University, Erlangen-Nürnberg (ID 19-426-B). All participants or their authorized legal representatives provided written informed consent after receiving a complete description of the study. The investigation was conducted according to the principles expressed in the Declaration of Helsinki.

### 2.5. Statistical Analysis

We present the data as means and standard deviations or relative frequencies. The CERAD-NB+ scores are presented as age-, sex- and education-adjusted z-scores calculated according to the CERAD-NB+ manual [69,72]. Levene’s test was used to evaluate the homogeneity of variances and normal distribution was checked with the Shapiro–Wilk’s test or visual examination of QQ-plots. The χ^2^ test was applied to assess differences in nominal, ordinal and non-normally distributed variables (e.g., depressiveness, living in a partnership, sex). Continuous variables were compared using *t*-tests, analyses of variance (ANOVA) or Welch ANOVA for heterogenous variances (e.g., age, level of education, CERAD-NB+ scores). Pearson’s correlations were used to investigate continuous relationships between the 2D:4D measures and CERAD-NB+ scores in the patient groups. R2D:4D and L2D:4D were compared, stratified by sex between the patient and control groups, using a 2 (sex) × 4 (group) MANOVA with two dependent variables (R2D:4D, L2D:4D). Prior to computing the MANOVA, the following assumptions were checked [73]: multivariate normality was tested with the multivariate Shapiro–Wilk’s test, homogeneity within the variance–covariance matrices was evaluated by applying the Box’s M test and multivariate outliers were identified by calculating the Mahalanobis distance for each observation. As the MANOVA test statistic, we used the Wilks’ Lambda Test Statistic [74]. Statistical significance was designated at *p* < 0.05 (two-sided). The data were analyzed using the statistical software R, version 4.1.1 [75]. Figures were generated using GraphPad Prism, version 9 (Graph Pad Software Inc., San Diego, CA, USA).

## 3. Results

### 3.1. Study Cohort Characteristics

The cohort consisted of 139 participants. We excluded 31 of these individuals due to the following reasons: 10 confounding neurological illnesses, 7 frontotemporal instead of Alzheimer’s dementia, 6 illnesses of the fingers, 4 unclear diagnoses, 3 healthy controls not being classified as “healthy” and 1 withdrawn consent. The remaining 108 participants consisted of 65 men and 43 women with a mean age of 67.7 ± 9.8 years and 69.2 ± 7.6 years, respectively. Of these, 30 participants were diagnosed with AD and 18 participants belonged to the tauopathy group; the clinical control group consisted of 31 participants and 28 subjects were classified as healthy controls. We exceeded our previously defined ideal sample size (*n* = 102). The participants’ sociodemographic characteristics, divided according to group allocation, are detailed in Table 1. Table 1 also displays the participants’ psychological well-being (operationalized via depressiveness [76,77] and SCL-scores [78,79,80]), their digit ratio and levels of AD-typical CSF biomarkers for patients and clinical controls. The groups are comparable regarding mean age, level of education and living in a partnership. As expected, they differed concerning depressiveness, with clinical controls reporting the highest depression scores and AD patients scoring the lowest MMSE scores. The distribution of men and women was unequal among the groups, with the highest percentage of males in the AD group and the lowest percentage of males among the clinical controls (Table 1).

### 3.2. Neuropsychological Examination

**Figure 1:**
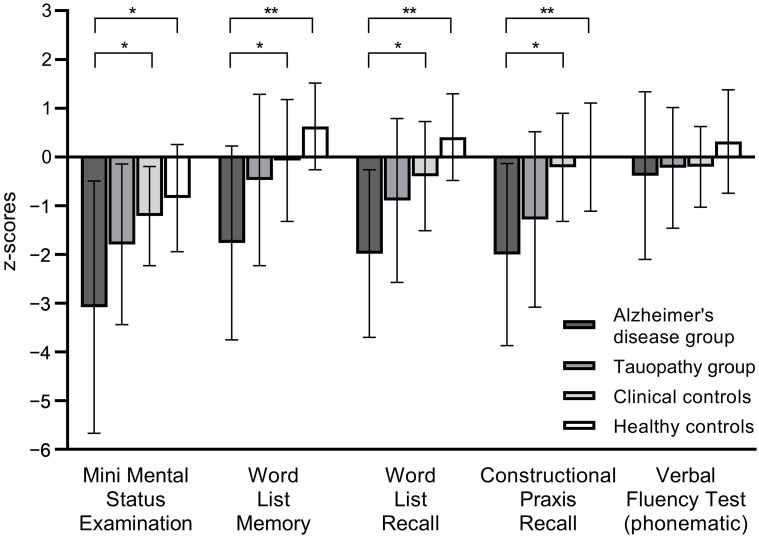
The groups’ performances (as z-scores with standard deviations) in the five CERAD neuropsychological battery PLUS tests of particular interest (Mini-Mental Status Examination, Word List Memory, Word List Recall, Constructional Praxis—Recall and Phonematic Verbal Fluency Test). Significant group differences are indicated with * (*p* < 0.05) and ** (*p* < 0.001).

The CERAD-NB+ was able to distinguish well between our four groups, with the AD group performing the worst and the healthy controls performing the best in all analyzed subtests. The CERAD-NB+ performances, split according to group allocation, are illustrated in Figure 1 and descriptive statistics are given in Supplementary Table S1. We did not find significant group differences in the Phonematic Verbal Fluency Test (F(3, 46) = 1.8, *p* = 0.160), but the study groups differed significantly in the MMSE (F(3, 42) = 5.5, *p* = 0.002) and the Word List Memory (F(3, 45) = 10.8, *p* < 0.001), Word List Recall (F(3, 46) = 13.8, *p* < 0.001) and Constructional Praxis Recall (F(3, 46) = 8.6, *p* < 0.001) tasks. The post hoc t-tests following the significant ANOVA results revealed significant differences between the AD group and the healthy and clinical controls for all four tests (Figure 1 and Supplementary Results).

### 3.3. Second-to-Fourth Digit Length Ratio

When analyzing common 2D:4D sex differences, both the R2D:4D and L2D:4D of males (right: 0.957 ± 0.02, left: 0.957 ± 0.02) and females (right: 0.968 ± 0.03, left: 0.965 ± 0.04) did not differ significantly between the sexes (right: t = −1.8, *p* = 0.079, left: t = −1.3; *p* = 0.209).

The results of the MANOVA comparing AD patients with all other groups (main and interaction effects) are detailed in Table 2; the sex-stratified means and standard deviations for all groups can be found in Supplementary Table S2. The assumption of multivariate normal distribution was met for both dependent variables (multivariate Shapiro–Wilk’s test *p* > 0.05). The Box’s M-test for homogeneity of covariance matrices revealed a homogenous matrix regarding “group” (χ^2^(9) = 12.01; *p* = 0.21) and “sex” (χ^2^(3) = 8.91; *p* = 0.03). Measuring the Mahalanobis distance for each observation indicated no multivariate outliers. As can be seen in Table 2, there is no significant difference between the sexes or between the groups for the combined dependent variables. Furthermore, no significant interaction between sex and group was detected. Effect sizes (partial η^2^) are in the small range.

Descriptively, we detected the largest difference between female clinical and healthy controls (Supplementary Table S2), with clinical controls revealing higher 2D:4D ratios for both hands. This was statistically significant for the right hand (R2D:4D: t = 2.33, *p* = 0.029, L2D:4D: t = 2.02, *p* = 0.054). Our female clinical controls were all at least once in their lifetime diagnosed with a depressive disorder. Therefore, we post hoc evaluated, in a subsequent exploratory analysis, whether the 2D:4D of female AD patients with concomitant late-onset depression also differed from healthy controls, which was not true in our sample (R2D:4D: t = −0.47, *p* = 0.644, L2D:4D: t = −0 0.76, *p* = 0.457). This analysis is limited due to the small sample size in the group of female depressive AD patients (*n* = 4) and is not sufficiently powered.

The results of the correlative analysis of 2D:4D and CERAD-NB+ scores in the patients’ groups (AD and tau groups) are presented in Supplementary Tables S3 and S4. In a sex-stratified Bonferroni correction, significance levels were adjusted at *p* = 0.003 (2 hands × 2 groups × 5 CERAD-NB+ tests). The nominally significant results we found did not survive Bonferroni correction. Again, power for the correlative analyses was low.

## 4. Discussion

In the present study, we examined possible associations between the 2D:4D digit ratio as a marker for prenatal androgen load and AD. In a biochemically and psychometrically well-characterized sample, we did not detect any significant differences in 2D:4D between AD patients and tauopathy patients or the control groups (clinical controls, healthy controls) and, thus, we did not confirm our hypotheses. Moreover, there was no significant relationship between 2D:4D and the performance of patients with AD or tauopathy in different memory tasks. Our results do not agree with previous studies on this topic in which the authors describe both higher [48] and lower [49,50] 2D:4D in female AD patients, higher 2D:4D in male AD patients compared with controls [49] and several associations with memory task performance [43,44]. Compared to these investigations, our study differs primarily in terms of sample characterization and diagnostic procedures. We applied multiple diagnostic methods, including a lumbar puncture with CSF analysis, cranial imaging methods, extensive neuropsychological characterization and multi-disciplinary diagnostic evaluations. As a consequence, our study group allocation was based on precise criteria, and we did not have to rely on diagnoses received from other practitioners or the participants’ self-reporting. Another difference from the existing literature is our 2D:4D measuring method. Hand scans were evaluated by three different raters instead of measuring the participants’ hands directly, as in Vladeanu et al. (2014) [49], or having only one person assess finger length, as in Jiang et al. (2020) [48]. This measurement technique is cost-effective, reliable and more independent of the subjects’ cooperation during the examination [81]. Furthermore, it facilitates blinding the raters since hand scans are more easily anonymized [82].

We could not confirm a difference between male and female 2D:4D in our sample, which is a common finding in digit ratio research [19,20,26]. Studies on 2D:4D among the elderly are rare and it has been speculated that the digit ratio slightly changes throughout life, for example, due to the dominant usage of one hand [83]. A preliminary investigation indicates that, for women, associations of 2D:4D and cognitive scores change as participants age; the turning point seems to be around 65 years old [84], which 77% of the women in our study exceeded. Another potential explanation might be the lower normalized life expectancy in men with lower 2D:4D [85], suggesting that our old male sample (mean age: 67.7 ± 9.8 years) might consist of the remaining males with more feminized digit ratios, thus deflating the 2D:4D sex difference. However, more studies are warranted to clarify the role of 2D:4D in later life.

Here, we identified a significant difference in the R2D:4D between female healthy and clinical controls, with women with symptoms of depression having a higher 2D:4D than healthy controls. This partly matches the existing literature on higher 2D:4D correlating with higher female depression scores [55] and higher 2D:4D in depressed female patients accompanied by hopelessness [86]. Nevertheless, the literature on 2D:4D and depression is ambiguous with, for example, studies also reporting null results for females but significant associations for males [53] and studies suggesting lower instead of higher digit ratio in depressed women [54]. In contrast to our clinical controls with early-onset depression (i.e., before the age of 60 years), we found in a post hoc exploratory analysis that the 2D:4D of female AD patients with concomitant late-onset depression did not differ from healthy controls. This corresponds to the assumption that early- and late-onset depression are of similar appearance but differ etiologically [87]. Our data might suggest a potential distinguishing role of 2D:4D between these two subforms of depressive disorders. However, this finding requires cautious interpretation due to the very small sample size in the AD group and the low power of this secondary analysis. Additionally, one has to bear in mind that the etiology of depression is multifactorial and cannot be narrowed down to one influencing factor (for a summary of etiological influences on depression among the elderly, see [88,89,90,91]). Further research is needed to clarify this possible association.

### Strengths and Limitations

In this study we provide reliable data of a well-characterized sample with precise diagnoses and met the required sample size of our a priori power analysis. We recruited two different control groups to be able to control for psychiatric illnesses and also to compare CSF-atypical to CSF-typical participants. All groups were comparable with respect to age, level of education and personal status. Additionally, the 2D:4D measurements were highly reliable, as indicated by the high ICCs. In contrast to previous studies in the field, 2D:4D was measured by multiple raters, and finger lengths were assessed with computer programs instead of calipers, which increase reliability [92,93]. Furthermore, measuring finger lengths indirectly with a scanner allows for blinded 2D:4D ratings.

There are some limitations to this study. First, there is criticism of the concept of 2D:4D as a biomarker in general, which has recently been summarized [94,95]: while Swift-Gallant et al. (2020) argued that the evidence of a consistent sex difference in 2D:4D, a masculinized ratio in CAH, a demasculinized ratio in Klinefelter’s syndrome and a feminized ratio in androgen insensitivity syndrome strongly indicate a correlation of 2D:4D with prenatal androgens [95], McCormick and Carré (2020) pointed out that statistical difficulties limit these interpretations [94]. Furthermore, studies suggest that mechanisms other than prenatal testosterone are involved in the development of 2D:4D, for example, prenatal stress [96] and prenatal corticosterone [97]. Second, men and women were disproportionally distributed among the groups (Table 1). The AD group consisted of mostly men, while the clinical control group mostly comprised women. This is a common pattern in the healthcare-seeking behavior of both sexes: Norcross et al. (1996) suggested that men in frail health are motivated by their wives to seek medical help [98]. In turn, subjects with mental health problems (here, allocated to the clinical control group) are more likely to seek psychiatric help when they are women [99]. Fourth, there are some methodological challenges inherent to the case–control study design, for example, selection bias when recruiting controls. In our sample, this might be a possible explanation for our healthy controls not differing from all other groups in burden by psychiatric or somatic symptoms (see Table 1). This influences the case–control difference and decreases the generalizability of results [100].

## 5. Conclusions

In our biochemically and psychometrically well-characterized sample, we did not detect any significant differences in 2D:4D between AD patients and tauopathy patients nor the control groups. This again emphasizes the necessity of applying reliable diagnostic and research instruments. We recommend a priori power analyses to determine the adequate sample size, to assess 2D:4D by multiple blinded raters and to define AD status using internationally standardized criteria. In general, more studies on 2D:4D in the elderly are needed to clarify whether changes in 2D:4D throughout the lifespan can be observed and whether current hormonal status in the elderly associates with 2D:4D. Additionally, studies are needed to clarify the role of 2D:4D in depression, with a focus on the differences between the early- and late-onset type.

## Figures and Tables

**Table 1:** Sociodemographic characteristics of the study sample.

	AD Group			Tau Group			Clinical Controls			Healthy Controls					
	*N*	M	SD	*N*	M	SD	*N*	M	SD	*N*	M	SD	F, χ^2^	*p*	η^2^
Men (%)	30	73.3		19	47.4		31	29.0		28	60.7		9.2	**0.026**	
Age (years)	30	71.6	10.8	19	70.4	8.5	31	62.7	5.7	28	69.6	7.6	0.2	0.653	0.002
Level of education (years)	26	14.8	3.3	19	13.8	2.8	31	14.8	2.6	28	15.7	3.0	3.9	0.272	0.008
Living in a partnership (%)	30	80.0		19	68.4		29	82.8		24	83.3		2.8	0.465	
Depressiveness ^a^ (%)	27	18.5		19	21.1		31	45.2		28	7.1		17.4	**0.043**	
Burden by psychiatric or somatic symptoms ^b^ (t)	28	55.7	7.2	18	55.8	7.8	31	59.9	8.7	28	50.0	10.0	1.2	0.277	0.011
MMSE score (z)	23	−3.1	2.6	16	−1.8	1.7	29	−1.2	1.0	28	−0.8	1.1	5.5	**0.003**	**0.138**
R2D:4D	27	0.962	0.02	18	0.955	0.02	30	0.966	0.03	27	0.962	0.03	1.0	0.310	0.010
L2D:4D	30	0.954	0.02	17	0.965	0.02	31	0.964	0.03	28	0.960	0.03	1.1	0.301	0.010
Aβ42 ^c^	29	609.96	235.88	18	1271.79	582.49	30	1025.90	328.37		NA		32.6	**<0.001**	**0.397**
Ratio Aβ42 to Aβ40 ^c^	29	0.0417	0.0098	18	0.0773	0.0151	30	0.0832	0.0118		NA		117.3	**<0.001**	**0.657**
pTau 181 ^c^	29	96.87	40.56	19	67.72	14.33	31	40.01	12.62		NA		49.6	**<0.001**	**0.619**
tTau ^c^	29	766.10	492.46	19	386.11	95.78	31	198.17	69.36		NA		56.0	**<0.001**	**0.701**

*p* < 0.05 in bold. ^a^ Percentage of subjects suffering from mild, medium or severe depressive symptoms. ^b^ Based on the t-scores in the global severity index of the Symptom Checklist 90-R [78] or of the corresponding short form SCL-K-9 [79]. ^c^ Cerebrospinal fluid markers were only available for patients and clinical controls. Aβ, Amyloid beta; AD, Alzheimer’s disease; MMSE, Mini-mental status examination; NA, not applicable; L2D:4D, 2D:4D of the left hand; pTau 181, phosphorylated tau at threonine 181; R2D:4D, 2D:4D of the right hand; tTau, total Tau.

**Table 2:** The MANOVA results of sex and study group (AD patients, tauopathy patients, clinical and healthy controls) on the combined R2D:4D and L2D:4D.

	Λ	F-Value	*df*1	*df*2	*p*-Value	η^2^_p_
Main effect of Sex	0.956	2.192	2	95	0.117	0.052
Main effect of Group	0.926	1.247	6	190	0.284	0.038
Interaction effect Sex × Group	0.911	1.510	6	190	0.177	0.045

AD, Alzheimer’s disease; *df*, degrees of freedom; Λ, Wilks’ Lambda Test Statistic; L2D:4D, 2D:4D of the left hand; R2D:4D, 2D:4D of the right hand; η^2^_p_, partial η^2^.

## Data Availability

The datasets generated during and/or analyzed during the current study are not publicly available due to data privacy restrictions but are available from the corresponding author on reasonable request.

## Supplementary Materials

The following supporting information can be downloaded at: https://www.mdpi.com/article/10.3390/brainsci13091229/s1, Supplementary Methods, Supplementary Results, Table S1: The CERAD-NB+ performances split according to group allocation (z-values); Table S2: The sex-stratified descriptive statistics of 2D:4D; Table S3: Sex-stratified Pearson correlations of 2D:4D and relevant CERAD-NB+ subtests in the Alzheimer’s group; Table S4: Sex-stratified Pearson correlations of 2D:4D and relevant CERAD-NB+ subtests in the tauopathy group.

## Note

The present work was conducted in partial fulfillment of the requirements for obtaining the degree “Dr. rer. biol. hum.” (Eva-Maria Siegmann).
