# Supplementary material for: Digit Ratio (2D:4D) Is Not Associated with Alzheimer’s Disease in the Elderly
Source: Brain Sci. 2023 Aug 22;13(9):1229. doi: 10.3390/brainsci13091229 (PMC10526128; doi:10.3390/brainsci13091229)
Supplement: Supplementary file 1 [file brainsci-13-01229-s001.zip › brainsci-2548147-supplementary.pdf]

Supplementary Information to

Digit ratio (2D:4D) is not associated with Alzheimer's disease in the elderly

Eva-Maria Siegmann[1,*], Pauline Olm[1], Bernd Lenz[2], Christiane Mühle[1], Timo Jan Oberstein[1], Juan Manuel Maler[1], and Johannes Kornhuber[1]

[1]Department of Psychiatry and Psychotherapy, Friedrich-Alexander University Erlangen-Nuremberg (FAU), Germany.

[2]Department of Addictive Behavior and Addiction Medicine, Central Institute of Mental Health (CIMH), Medical Faculty Mannheim, Heidelberg University, Germany.

*Corresponding author at: Department of Psychiatry and Psychotherapy, Friedrich-Alexander University Erlangen-Nürnberg (FAU), Schwabachanlage 6, D-91054 Erlangen, Germany, Phone: +49 9131 85-34166, E-Mail: eva-maria.siegmann@uk-erlangen.de

Supplementary Methods

Participants filled in psychological questionnaires to assess depressive symptoms with the German Beck's depression inventory II (BDI-II) (1) or the German short form of the Geriatric depression scale (GDS-15) (2). In this study, *depressiveness* was defined as exceeding the validated cutoff values of these questionnaires at the moment of study conduction. To screen for other psychiatric problems, healthy controls completed the German Symptom Checklist-90 Revised (SCL-90-R) (3) and the other groups answered the corresponding German short form SCL-K-9 (4, 5). The presence of a concomitant late-onset depression, defined as a depression diagnosis occurring after 60 years old, was evaluated post-hoc among the AD patients.

Literature

1. Hautzinger M, Keller F, Kühner C. Das Beck Depressionsinventar II. Deutsche Bearbeitung und Handbuch zum BDI II. Frankfurt: Harcourt Test Services; 2006.

2. Gauggel S, Schmidt A, Didie M. Der Einfluß von körperlichen Beschwerden auf die Diagnostik depressiver Störungen bei älteren Menschen. Zeitschrift für Gerontopsychologie und -psychiatrie 1994; (7):203–10.

3. Franke GH. SCL-90-R. Die Symptom-Checkliste von Derogatis - Deutsche Version - Manual. 2., vollständig überarbeitete und neu normierte Auflage. Göttingen: Beltz; 2002.

4. Klaghofer R, Brähler E. Konstruktion und Teststatistische Prüfung einer Kurzform der SCL-90–R. Zeitschrift für Klinische Psychologie, Psychiatrie und Psychotherapie 2001; (49):115–24.

5. Petrowski K, Schmalbach B, Kliem S, Hinz A, Brähler E. Symptom-Checklist-K-9: Norm values and factorial structure in a representative German sample. PLoS One 2019; 14(4):e0213490.

Supplementary Results

The post-hoc t-tests following the significant Welch ANOVA on group differences in the Word List Memory task revealed that this result is based on differences between the AD group and the clinical controls ($t = 1.7$, $p = .005$) as well as between the AD group and the healthy controls ($t = 2.4$, $p < .001$). The same holds true for the task Word List Recall with significant post-hoc differences between AD and clinical control ($t = 1.6$, $p = .002$) or healthy control subjects ($t = 2.4$, $p < .001$). Again, when examining post-hoc group differences in the task Constructional Praxis Recall we found them in the comparison AD patients versus clinical controls ($t = 1.8$, $p = .001$) and AD patients versus healthy controls ($t = 2.0$, $p < .001$). In the MMSE, the significant omnibus test can once again be traced back to significant group differences between the AD patients and the clinical control group ($t = 1.9$, $p = .015$) as well as the healthy control group ($t = 2.2$, $p = .003$).

Supplementary Table S1. The CERAD-NB+ performances split according to group allocation (z-values).

	AD group			Tauopathy group			Clinical controls			Healthy controls		
	N	M	SD	*N*	M	SD	*N*	M	SD	*N*	M	SD
Mini Mental Status Examination	23	-3.08	2.59	16	-1.79	1.65	29	-1.21	1.02	28	-0.84	1.10
Word List Memory	24	-1.76	1.99	18	-0.47	1.76	31	-0.07	1.25	28	0.63	0.89
Word List Recall	24	-1.98	1.72	18	-0.89	1.68	31	-0.39	1.25	28	0.41	0.89
Constructional Praxis - Recall	24	-2.00	1.87	18	-1.28	1.80	31	-0.21	1.11	28	0	1.11
Verbal Fluency Test (Phonematic)	24	-0.38	1.72	18	-0.22	1.24	31	-0.20	0.83	28	0.32	1.06

AD, Alzheimer's disease.

Supplementary Table S2. The sex-stratified descriptive statistics of 2D:4D

	AD group			Tau group			Clinical controls			Healthy controls		
	N	M	SD	*N*	M	SD	*N*	M	SD	*N*	M	SD
Men												
R2D:4D	20	0.963	0.02	9	0.945	0.02	21	0.954	0.02	11	0.964	0.02
L2D:4D	22	0.955	0.02	8	0.961	0.02	22	0.955	0.02	11	0.963	0.02
Women												
R2D:4D	7	0.959	0.03	9	0.965	0.03	9	0.993	0.04	16	0.960	0.03
L2D:4D	8	0.951	0.03	9	0.968	0.03	9	0.988	0.04	17	0.957	0.04

AD, Alzheimer's disease; L2D:4D, 2D:4D of the left hand; R2D:4D, 2D:4D of the right hand.

Supplementary Table S3. Sex-stratified Pearson correlations of 2D:4D and relevant CERAD-NB+ subtests in the Alzheimer's group

		Men			Women		
		N	r	*P*	*N*	r	*P*
MMSE	R2D:4D	20	-0.611	**0.016**	8	-0.722	0.105
	L2D:4D	22	-0.206	0.445	8	-0.838	**0.018**
Word List Memory	R2D:4D	20	-0.394	0.131	8	-0.831	**0.040**
	L2D:4D	22	-0.460	0.063	8	-0.915	**0.004**
Word List Recall	R2D:4D	20	-0.278	0.297	8	-0.116	0.826
	L2D:4D	22	-0.331	0.195	8	-0.313	0.494
Constructional Praxis Recall	R2D:4D	20	-0.571	**0.021**	8	-0.294	0.571
	L2D:4D	22	-0.153	0.558	8	-0.430	0.336
Phonematic Verbal Fluency Test	R2D:4D	20	-0.107	0.694	8	-0.800	0.056
	L2D:4D	22	-0.232	0.371	8	-0.850	**0.015**

$P < 0.05$ in bold (Bonferroni-corrected $P = 0.003$). L2D:4D, digit ratio of the left hand; MMSE, Mini Mental State Examination; R2D:4D, digit ratio of the right hand.
Annotation: The inferential findings in the female analysis are limited due to the small sample size.

Supplementary Table S4. Sex-stratified Pearson correlations of 2D:4D and relevant CERAD-NB+ subtests in the tauopathy group

		Men			Women		
		N	r	*P*	*N*	r	*P*
MMSE	R2D:4D	9	-0.616	0.141	9	0.314	0.449
	L2D:4D	8	0.342	0.507	9	0.434	0.282
Word List Memory	R2D:4D	9	-0.292	0.482	9	0.541	0.133
	L2D:4D	8	0.282	0.540	9	0.693	**0.039**
Word List Recall	R2D:4D	9	-0.419	0.301	9	0.563	0.115
	L2D:4D	8	-0.092	0.845	9	0.708	**0.033**
Constructional Praxis Recall	R2D:4D	9	-0.422	0.298	9	0.481	0.190
	L2D:4D	8	0.262	0.570	9	0.615	0.078
Phonematic Verbal Fluency Test	R2D:4D	9	-0.244	0.560	9	0.488	0.183
	L2D:4D	8	-0.019	0.968	9	0.519	0.153

$P < 0.05$ in bold (Bonferroni-corrected $P = 0.003$). L2D:4D, digit ratio of the left hand; MMSE, Mini Mental State Examination; R2D:4D, digit ratio of the right hand.
Annotation: The inferential findings in this table are limited due to the small sample sizes.
